# Supplementary figures and images for: Polyploidy-associated paramutation in Arabidopsis is determined by small RNAs, temperature, and allele structure
Source: PLoS Genet. 2021 Mar 9;17(3):e1009444. doi: 10.1371/journal.pgen.1009444 (PMC7978347; doi:10.1371/journal.pgen.1009444)

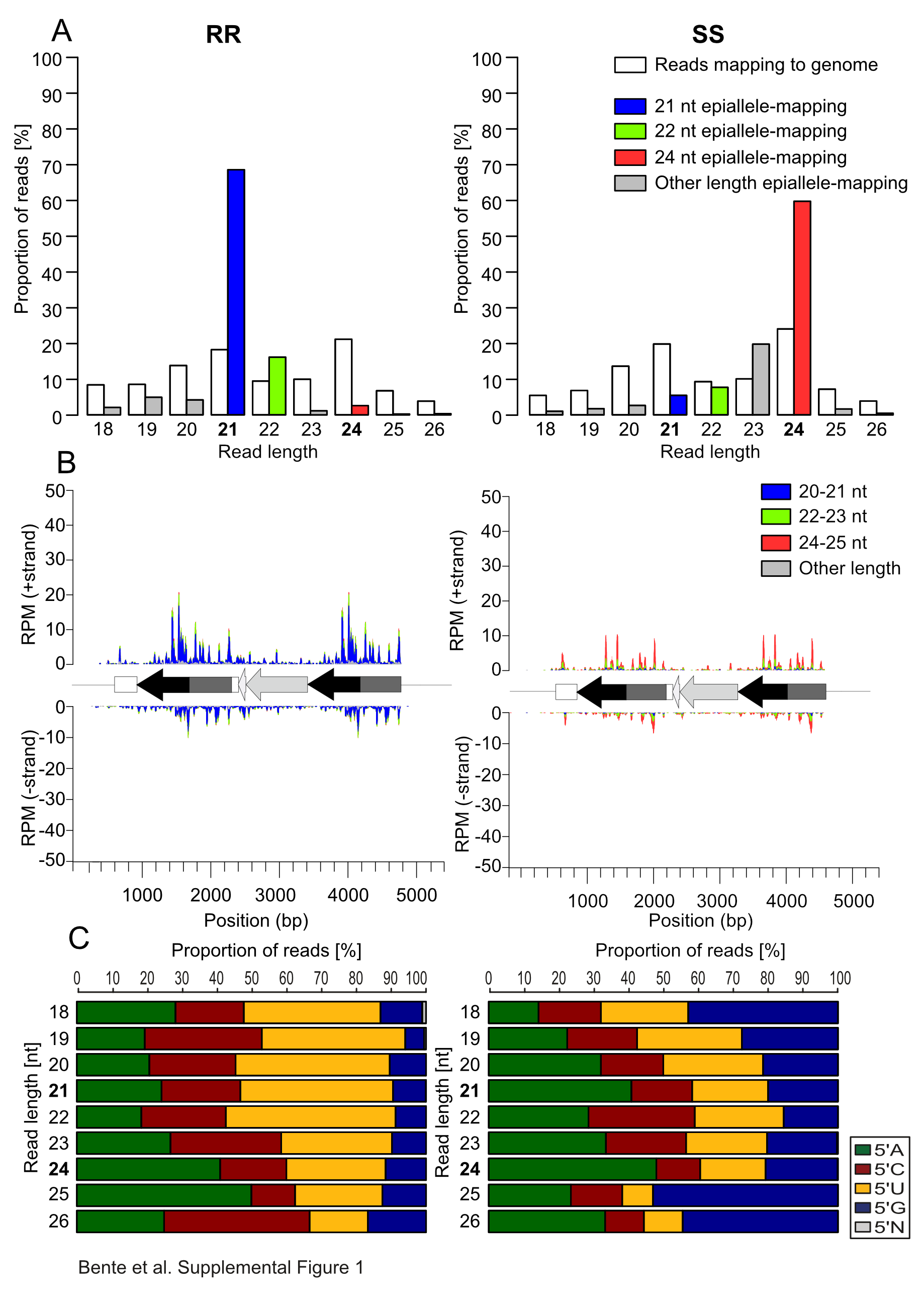

Supplement: S1 Fig — (A) Proportion of sRNA length from all mapped reads in 14 day-old seedlings with diploid active (RR) or silenced (SS) epialleles. (B) Coverage plots of 18–26 nt sRNA along the RR or SS epialleles. (C) Proportion of 5’ prime nucleotides of epiallele-specific sRNAs in RR (left) and SS (right) plotted by size. (TIFF) [file pgen.1009444.s001.tiff]

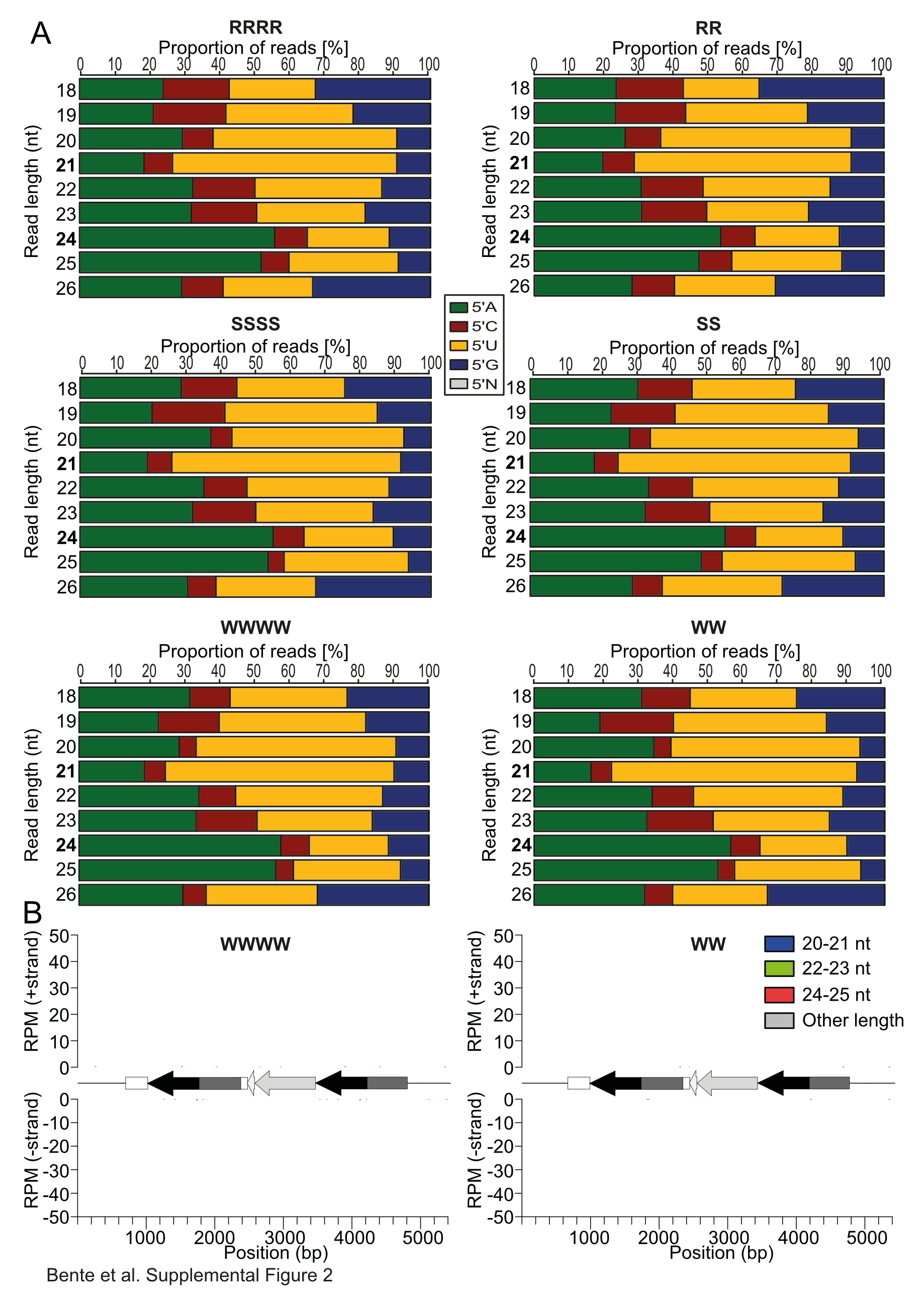

Supplement: S2 Fig — (A) Proportions of 5’ nucleotides in different size classes in sRNA libraries from tetraploids (left) and diploids (right) in lines with active epialleles (top), silent epialleles (middle), and wildtype lines (bottom). (B) RNA libraries from tetraploid (WWWW, left) or diploid (WW, right) plants were attempted to map to the full-length sequence of the epiallele. (TIFF) [file pgen.1009444.s002.tiff]

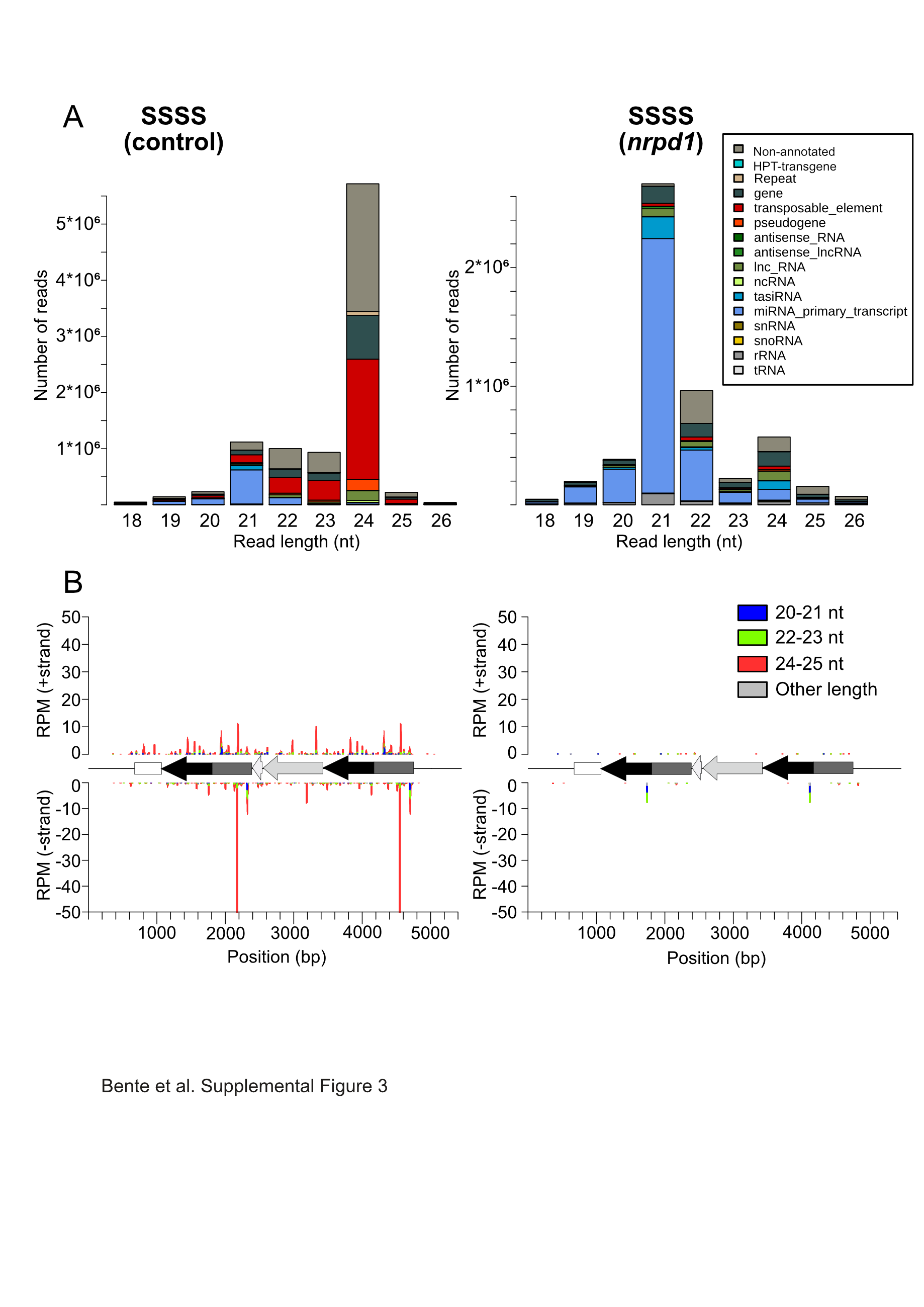

Supplement: S3 Fig — (A) Strong reduction of 24 nt sRNAs in a homozygous loss-of-function mutation in the NRPD1 gene (encoding the largest subunit of PolIV) generated by CRISPR in the background of the tetraploid silent epiallele; including (B) those mapping to the epiallele. (TIFF) [file pgen.1009444.s003.tiff]

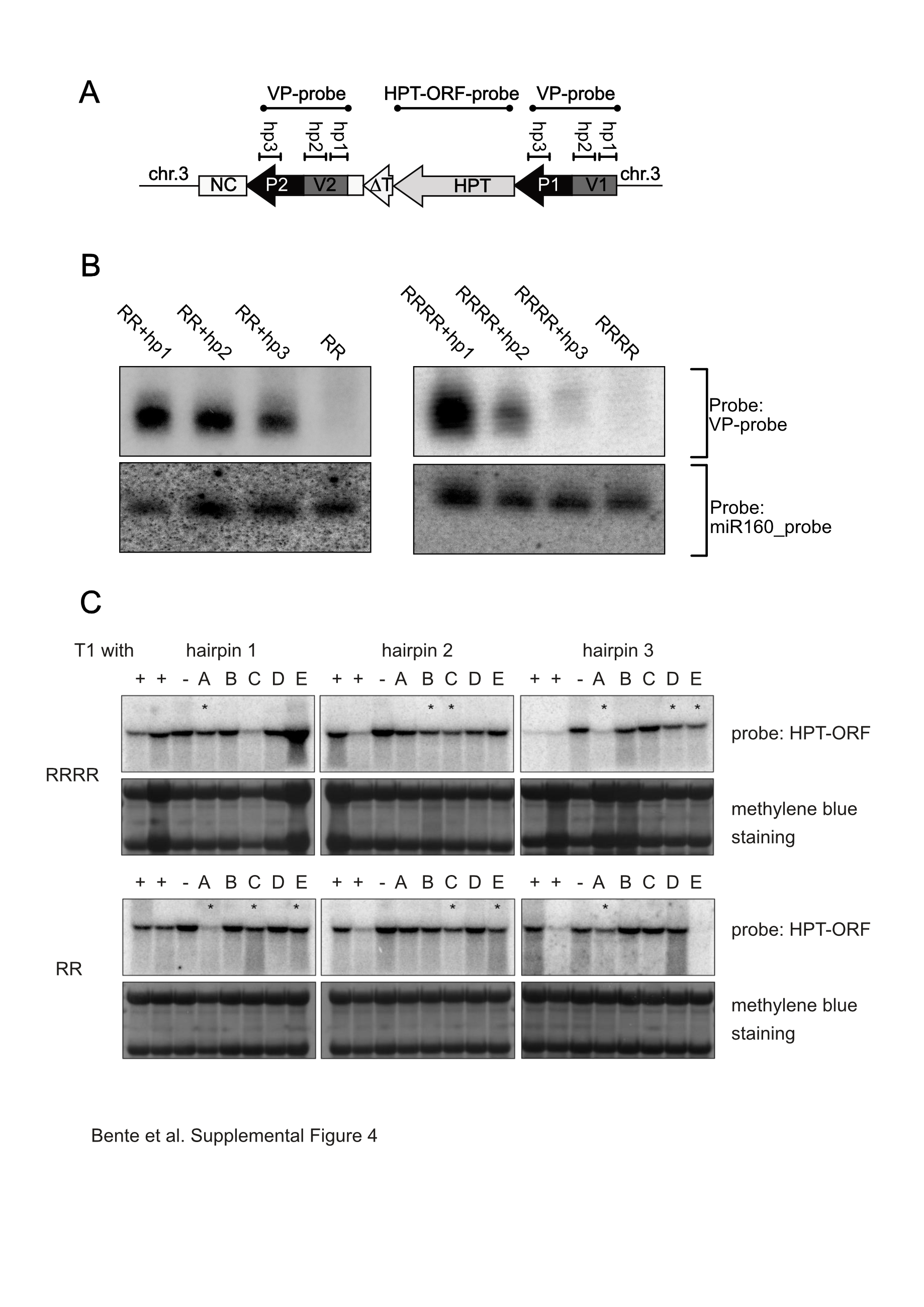

Supplement: S4 Fig — (A) Location of the hairpin sequences and region covered by the VP- and HPT-ORF probe for detection in B and C. (B) Northern blots with sRNA from flower buds of 35 d-old diploid and tetraploid T1 plants selected to contain the hairpin constructs. VP probe (labelled by random priming, top) and antisense miR160 (end-labelled oligonucleotide) as loading control (bottom). (C) Northern blot analysis of HPT transcript from tetraploid (top) or diploid (bottom) plants with R epialleles never containing the hairpin (-), individual T2 plants containing the hairpin (+) or not containing it any more due to segregation (A-E). Five μg of total flower bud RNA from 35 d-old plants was hybridized with the HPT-ORF or stained with methylene blue as loading control. Asterisks mark samples with reduced HPT transcript despite the absence of the hairpin. (TIFF) [file pgen.1009444.s004.tiff]

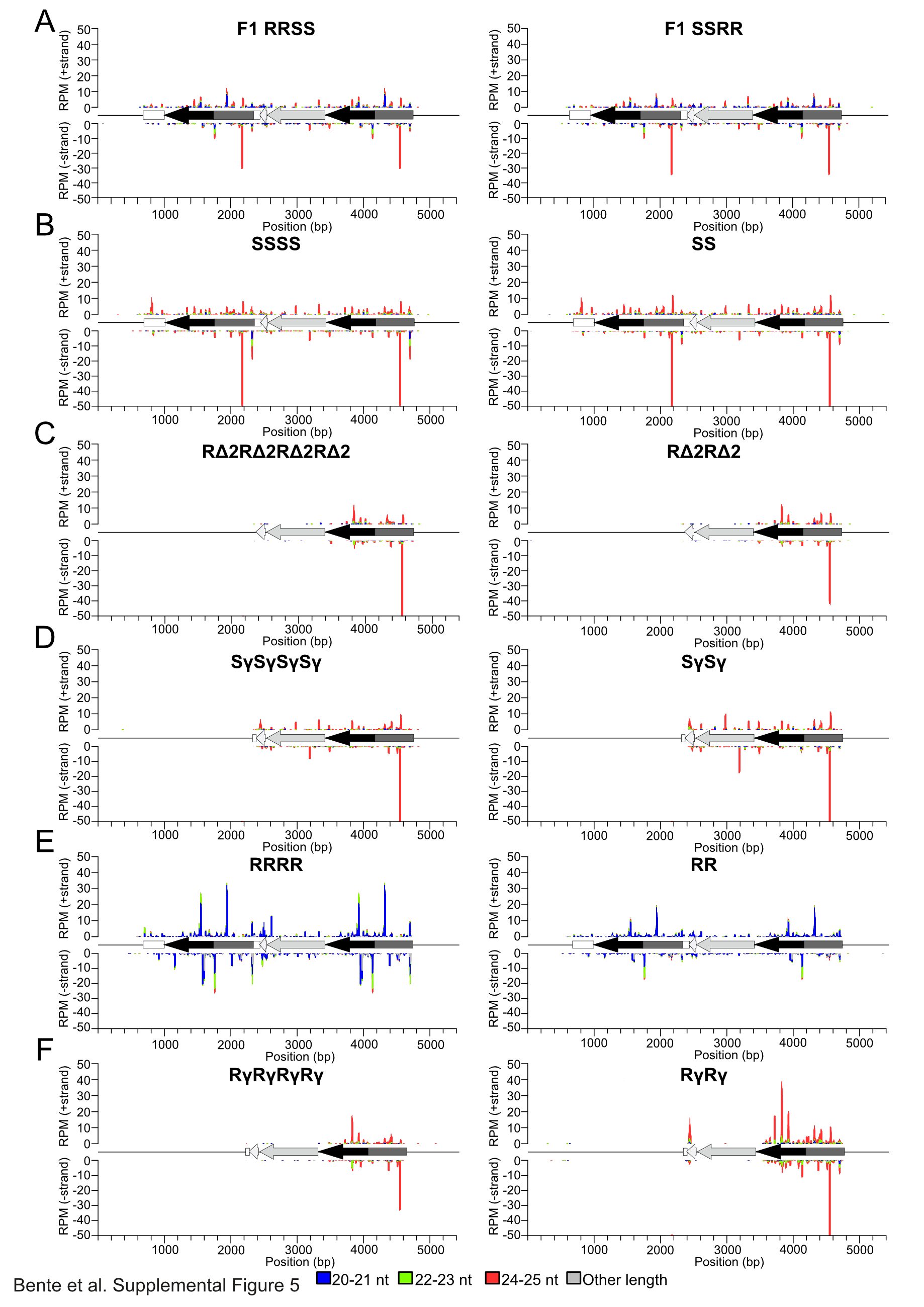

Supplement: S5 Fig — (A) Profile from flower bud material in a pair of reciprocal paramutation test hybrids. (B–F) Profiles of tetraploid (left) and diploid (right) lines with the indicated alleles; (B) silent full length allele; (C) active allele missing downstream repeat after random mutagenesis and hygromycin screen; (D) CRISPR-generated deletion of the downstream repeat in the background of the silent epiallele; (E) active full-length allele; (F) CRISPR-generated deletion of the downstream repeat in the background of the active epiallele. All values are reads per million mapped reads (RPM), and the y-axes are all set to the same maximum of ± 50 RPM. (TIFF) [file pgen.1009444.s005.tiff]

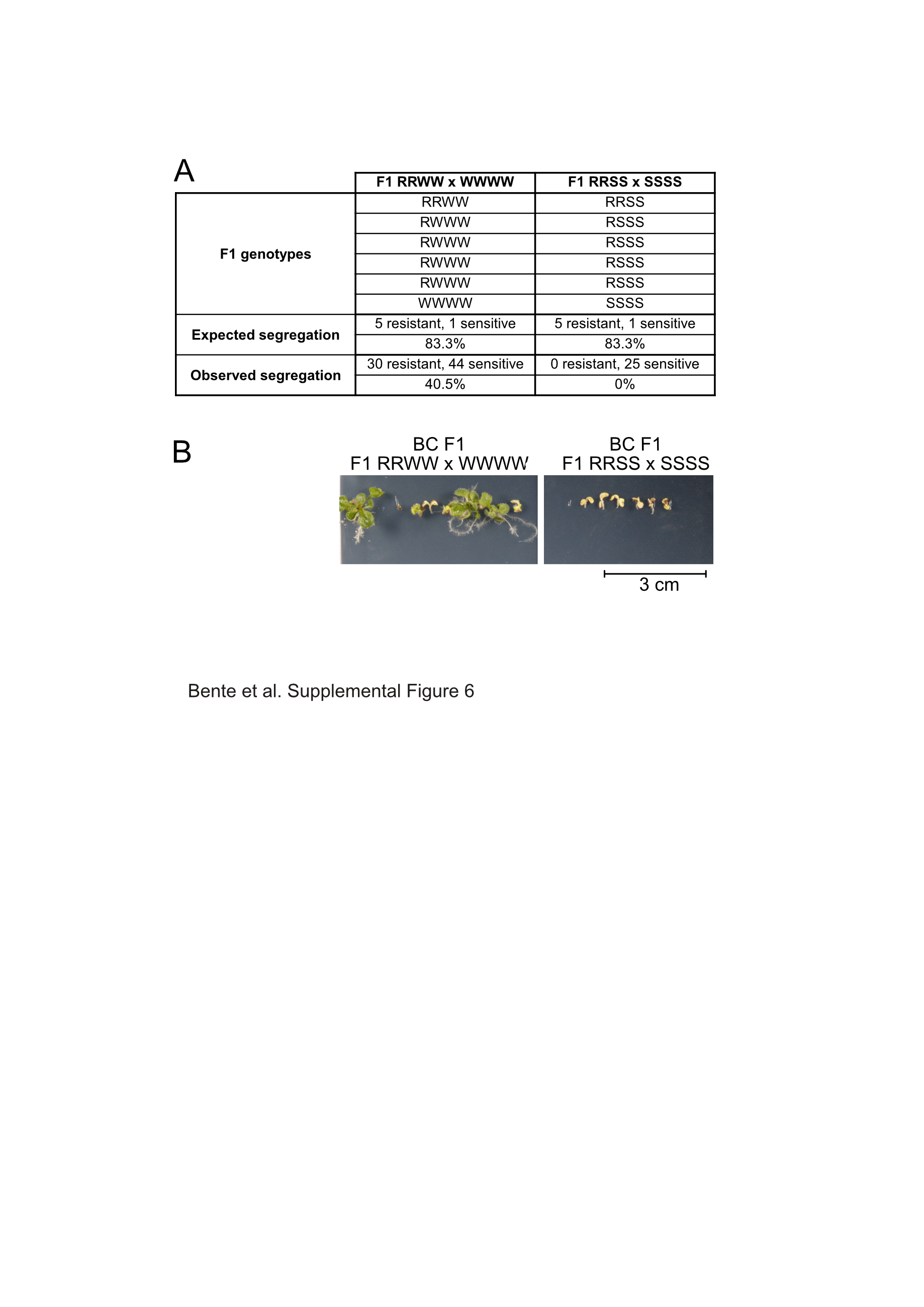

Supplement: S6 Fig — (A) Tetraploid hybrids with two alleles each of R and W, or R and S, were backcrossed to homozygous W or S, respectively. In both cases, two thirds of the F1 progeny will contain one R epiallele, and 5 out of 6 plants can be expected to be resistant. The lack of any resistant plants in the crosses involving S indicates a strong silencing effect in case of three S in combination with one S. (B) Representative picture of three-week-old plants from the indicated genotypes grown on hygromycin selection medium. Scale bar = 3 cm. (TIFF) [file pgen.1009444.s006.tiff]
